# Effect of Process Parameters and Material Properties on Laser Micromachining of Microchannels

**DOI:** 10.3390/mi10020123

**Published:** 2019-02-14

**Authors:** Matthew Benton, Mohammad Robiul Hossan, Prashanth Reddy Konari, Sanjeewa Gamagedara

**Affiliations:** 1 Department of Engineering and Physics, University of Central Oklahoma, Edmond, OK 73034, USA; matthewjbenton@outlook.com (M.B.); pkonari@uco.edu (P.R.K.); 2 Center for Interdisciplinary Biomedical Education and Research, University of Central Oklahoma, Edmond, OK 73034, USA; sgamagedara@uco.edu; 3 Department of Chemistry, University of Central Oklahoma, Edmond, OK 73034, USA

**Keywords:** laser micromachining, laser ablation, microchannels, microfabrication, laser system parameters, modeling of laser micromachining

## Abstract

Laser micromachining has emerged as a promising technique for mass production of microfluidic devices. However, control and optimization of process parameters, and design of substrate materials are still ongoing challenges for the widespread application of laser micromachining. This article reports a systematic study on the effect of laser system parameters and thermo-physical properties of substrate materials on laser micromachining. Three dimensional transient heat conduction equation with a Gaussian laser heat source was solved using finite element based Multiphysics software COMSOL 5.2a. Large heat convection coefficients were used to consider the rapid phase transition of the material during the laser treatment. The depth of the laser cut was measured by removing material at a pre-set temperature. The grid independent analysis was performed for ensuring the accuracy of the model. The results show that laser power and scanning speed have a strong effect on the channel depth, while the level of focus of the laser beam contributes in determining both the depth and width of the channel. Higher thermal conductivity results deeper in cuts, in contrast the higher specific heat produces shallower channels for a given condition. These findings can help in designing and optimizing process parameters for laser micromachining of microfluidic devices.

## 1. Introduction

Microfluidic devices have been shown to have a broad range of potential applications in chemistry, biology, engineering, and biomedical sciences such as polymerase chain reaction (PCR) [1,2,3], protein separation and analysis [4,5,6], sample mixing [7], diagnostics [8], and chemical reactions [7,9] etc. The use of microfluidics offers several competitive advantages such as requiring smaller sample volumes, fewer reagents, greater portability, faster processing, easier disposability, and better compatibility with smart devices [8]. However, mass production of these devices without compromise on performance is an ongoing challenge.

Photolithography, chemical etching, soft lithography, hot embossing and injection molding are the most commonly used microfabrication processes for fabricating microfluidic devices [10]. Many of these processes are either expensive, incompatible for mass production, or limited to use on certain materials as substrates [11,12,13]. For instance, chemical etching and photolithographic process mainly use silica based materials such as silicon wafer, glass and polydimethylsiloxane (PDMS) as substrates [2]. Recently laser ablation has emerged as a promising technique for rapid fabrication of microfluidic devices [14]. Laser based fabrication provides distinct advantages over these traditional fabrication techniques, including processing speed, quality, flexibility, and scalability.

Laser ablation is a process of removing material from the surface of an object using an intensely focused electromagnetic radiation. This material removal process depends on the fluence of the laser, material properties and types of laser [15,16]. The use of both pulsed ultraviolet (UV) and continuous infrared (IR) lasers have been demonstrated for microfabrication. In the case of UV fabrication, high energy light is used to induce a highly targeted breakdown of materials in very short pulses [15,17]. The short pulse laser interaction with polymeric materials are discussed in details by Kruger and Kautek [18]. However, UV lasers are not widely used in microfluidic fabrications due to expensive, complicated processes and low repetition rate [19]. On the other hand, the IR laser systems, mainly in the form of CO_2_ lasers, are comparatively simple and more popular in industrial production [20]. Their use in microfluidics was first demonstrated by Klank et al. [21], who used an industrial CO_2_ laser as an effective alternative to UV laser systems for fabricating microfluidic devices in polymer materials such as poly-methylmethacrylate (PMMA).

Since then, there have been a handful of reports on the CO_2_ laser machined microfluidic devices for various biomedical applications [19]. Liu et al. [1] used a CO_2_ laser to create disposable integrated devices for genetic assay in polycarbonate. The device successfully integrated PCR amplification, DNA hybridization, and hybridization wash functions. Wang et al. [22] created a microfluidic device using CO_2_ laser on Vivak co-polymer for electroosmotic pumping and electrophoretic separations without any surface modification. A selective hydrophobic and hydrophilic surface in laser machined PMMA microchannel was demonstrated by Wang and co-workers through modulation of laser parameters [23]. Yang et al. [24] reported using high throughput CO_2_ laser ablated microfluidic device for chitosan microparticles encapsulation of ampicillin. Similar CO_2_ laser ablated microfluidic devices were used in other microfluidic applications [25,26]. Despite these reports, one barrier to widespread use of laser ablated microfluidic channels is that they commonly suffer from architectural defects [27,28].

Bulge formation, surface roughness, and irregular profile of the channels are common issues found in laser fabricated microfluidic devices, regardless of the substrate material used [28]. To address this concern, several techniques have been investigated that can minimize these drawbacks. Hong et al. [25] reported that using an unfocused CO_2_ laser beam resulted in reduced roughness. Cheng et al. [29] reported that the use of a thermal annealing process after channel fabrication reduced average roughness from 5–10 µm to around 2–3 nm in PMMA channels. They also found that the use of extruded PMMA in microchannel fabrication reduced roughness, though more bulge formation was observed [29]. The opposite trend was reported for cast PMMA, where roughness was more pronounced, but negligible rim distortion was observed [29]. Recently Ahmed et al. [30] demonstrated that the use of fluence and pulses-per-spot (F-PPS), and the accumulated fluence profile (AFP) models with conjunction of laser parameters produced controlled surface topography. Another report suggests that combined treatment of femtosecond and CO_2_ laser machining improves surface smoothness [31].

Though numerous experimental studies on the laser based fabrication of microfluidic devices have been conducted, there are only a handful of studies that focus on a theoretical investigation of laser micromachining. Snakenborg et al. [20] presented a model to predict the depth of cut by a CO_2_ laser in PMMA by estimating the amount of absorbed heat. Tresansky et al. [32] used a finite element based 2D axisymmetric model of polymer to determine the removal of material during laser ablation. They used a material phase dependent thermal conductivity in their model and simplified the model to avoid re-meshing at each step. Similar analyses were also reported in [14]. However, to the best of our knowledge, there are no comprehensive numerical studies that relate laser systems parameters and thermos-physical properties of the substrate to characterize the channel geometry i.e., depth, profile, and width of the channel. This research studied the effect of both material properties and laser system parameters on the shape, width and depth of the microchannel. A three-dimensional computational model was developed using finite element based Multiphysics software COMSOL 5.2a (Burlington, MA, USA). The transient heat conduction equation was solved considering a continuous, Gaussian laser beam as a moving heat source. The effect of laser spot size, laser scanning speed and laser power on the resulting channel width, depth, and shape were investigated. A parametric study of the effect of various relevant thermos-physical properties of the substrate materials was also investigated. The rest of the paper is organized as follows: Section 2 contains theory, model formulation, boundary conditions, convergence studies; Section 3 provides results and discussion; and finally, Section 4 provides conclusions of the study.

## 2. Theory and Methods

### 2.1. Theory

When a continuous IR laser beam, such as the CO_2_ laser, is moved across the surface of a polymer, the laser energy is absorbed as heat, which rapidly increases the temperature of the material in the irradiated region. At the spot where the laser meets the surface, a portion of the material is vaporized, while another portion melts into a liquid form. The vaporized material expands and escapes, driving the liquid polymer outward along with it. In this way, some of the liquid material is ejected, while the rest remains in the channel and re-solidifies in the wake of the beam. By moving the laser across the surface, a channel and other similar structures can be cut into the material. The cross section of the resulting channel depends on the material which is mostly regulated by thermal diffusivity of that material, as well as various laser parameters. Therefore, the CO_2_ laser micromachining of microfluidic channels can be modeled as a thermal event [33] using the transient heat equation.

Although the material undergoes a phase change as it is heated by the laser, most of the heating process occurs in the solid phase. Once the material reaches the point of phase change, it quickly changes phase and is removed from the bulk solid material. Therefore, the phase change and subsequent material removal can be modeled as a convection boundary condition in a transient heat conduction equation with laser irradiation as a moving heat source. 

### 2.2. Governing Equations

**Figure 1:**
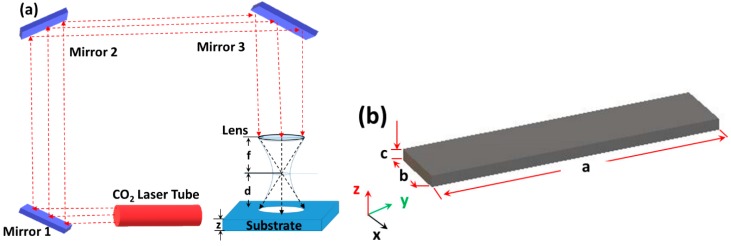
(**a**) Schematic of a typical CO_2_ laser machining setup, where *f* is the focal length of the focusing lens, and *d* is the distance between the substrate and the focusing length. (**b**) Schematic of a polymer slab that is subject to laser ablation. The dimensions (a × b × c) of the slab are parametrically defined based on the size of the laser beam and channel length.

To model laser cutting as a thermal event, the transient heat equation is given by [34]:

(1) $$\rho C_{p}\frac{dT}{dt}=\vec{\nabla }\cdot \left(k\vec{\nabla }T\right)+{\dot{q}}_{v}$$

where *ρ* is the density, *C_p_* is the constant pressure specific heat, and *k* is the thermal conductivity of the material. The ${\dot{q}}_{V}$ term represents volumetric heat generation due to the absorption of the laser beam at the surface. The specific laser energy absorption is given by using Beer’s Law [35]:

(2) $${\dot{q}}_{v}=\alpha I\left(r,z\right)e^{-\alpha z}$$

where *α* is the material’s absorption coefficient (m^−1^) and *I(r,z)* is the intensity of the laser at a distance *r* from the axis of the beam, the *z*-coordinate is the distance from the surface of the substrate measured downwards in the direction of the beam. The intensity profile of the laser beams is given by a Gaussian function [36]:

(3) $$I\left(r,z\right)=I\left(0,z\right)e^{-\frac{2r^{2}}{w{\left(z+d\right)}^{2}}}$$

where *I*(0,*z*) is the peak intensity along the beam axis, $I\left(0,z\right)=\frac{2P_{0}}{\pi w{\left(z+d\right)}^{2}}$, *P_0_* is the total power of the laser beam, *w(z)* is the beam radius, or spot size, at which point the beam intensity falls to 1/*e*^2^ of its value along the beam axis, and *d* is the distance from the substrate to the focal point of the focusing lens as shown in Figure 1a.

The size of the beam, described by the beam radius, is given by the following function:

(4) $$w\left(z\right)=w_{0}\sqrt{1+{\left(\frac{\lambda z}{\pi w_{0}^{2}}\right)}^{2}}$$

where *λ* is the wavelength of the laser and *w*_0_ is the waist radius, the minimum radius of the laser beam. Assuming the beam radius is approximately constant over relevant *z* values, the beam radius becomes a constant *w*_0_. Combining and simplifying Equations (2)–(4), and using *r*^2^ = *x*^2^ + *y*^2^,

(5) $${\dot{q}}_{v}=\frac{2p_{0}\alpha }{\pi w_{0}^{2}}e^{-\frac{2\left(x^{2}+y^{2}\right)}{w_{0}^{2}}}$$

For laser cutting, the laser beam is moved continuously across the surface. Assuming the laser beam moves at a constant velocity, $v_{L}$ along the x-coordinate direction, the time dependent power density can be written as:

(6) $${\dot{q}}_{v}=\frac{2p_{0}\alpha }{\pi w_{0}^{2}}e^{-\frac{2(\left(x+v_{L}t{)}^{2}+y^{2}\right)}{w_{0}^{2}}-\alpha z}$$

### 2.3. Boundary Conditions

All boundaries and surfaces except the top surface where the laser beam impinges are treated as thermally insulated. The boundary conditions for those surfaces and boundaries are given by

(7) $$k\vec{\nabla }T\cdot \hat{n}=0$$

where $\hat{n}$ is the unit normal vector at the boundary. The top surface where laser irradiates is represented with a combination of radiation and convection heat transfer boundary condition as follows:

(8) $$-k\vec{\nabla }T\cdot \hat{n}=h_{c}\left(T_{\infty }-T_{s}\right)+\varepsilon \sigma \left(T_{\infty }^{4}-T_{s}^{4}\right)$$

where *h_c_* is the convective heat transfer coefficient, $T_{s}$ is the surface temperature, *ε* is the emissivity of the material, and *σ* is the Stefan-Boltzmann constant. The choice of the convection coefficient is described in detail in the numerical modeling section.

### 2.4. Numerical Simulation

A three dimensional rectangular slab of PMMA was considered to perform numerical simulation in COMSOL 5.2a as shown in Figure 1a. The dimensions of the rectangular slabs were parametrically defined based on the size of the laser beam and channel length to ensure that the region affected by heat is entirely contained within the model. 

The time dependent heat equation (Equation (1)), was solved for the domain shown to find the temperature distribution by the laser. The PARDISO, an efficient robust direct solver with backward differentiation formula (BDF) time stepping method, was employed. The temperature distribution was then used to determine where the material was removed. The material was assumed to be removed once the material reached the temperature at which phase change from solid to vapor is complete. Although the phase change and material removal for a polymer such as PMMA will occur over a range of temperatures, the heating is assumed to happen rapidly enough that it can be approximated as a step change between solid and vapor phases at temperature, *T* = 700 K for PMMA [32].

In this study, a convective heat transfer coefficient was used to model phase change and the heat loss associated with the laser ablation. At the melting phase, heat convection occurs at the solid-liquid boundary as well as between the liquid and the surrounding air. Once the liquid begins to vaporize, it boils out, quickly expands, and escapes with large amounts of heat due to convection. This phenomenon resembles pool boiling heat transfer. In pool boiling, boiling occurs on the solid-liquid interface when the solid surface temperature exceeds the saturation temperature of the liquid. The phase transition in pool boiling results in very high levels of convective heat transfer [34]. Therefore, a very high convective heat transfer coefficients on the order of 10^5^ W m^−1^ K^−1^ was used in order to model laser micromachining in this study. A range of heat transfer coefficients was explored. 

### 2.5. Convergence Study

**Figure 2:**
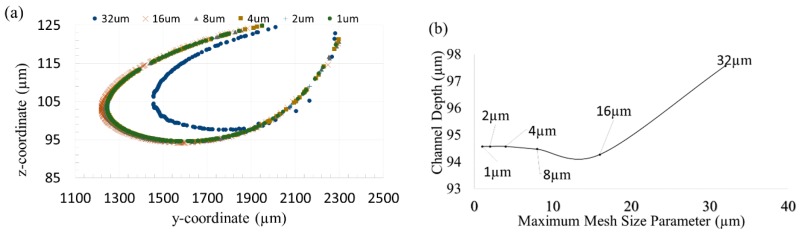
The temperature and depth of cut as measured at *t* = 0.0052 s for different mesh sizes for convergence analysis. Constant material properties and laser system parameters listed in Table 1 and Table 2 were used. (**a**) *T* = 700 K Isotherm for different element sizes; (**b**) Channel depth determined from each mesh.

Second order unstructured tetrahedral mesh elements were used to discretize the domain. Convergence analysis was conducted in order to determine the optimal element size to use. Temperature and depth of cut were used as convergence study parameters. Using constant parameters, which are listed in Table 1 and Table 2, the location of the *T* = 700 K isotherm in the domain was recorded at a given time for element sizes ranging from 32 µm (coarse) to 1 µm (very fine) as shown in the Figure 2a. It was found that the profile of the isotherm varied negligibly for element sizes 4 μm and lower. The depth of cut, reported as the largest depth at which temperature reaches removal temperature, was also found to not vary significantly for element sizes 4 μm or lower, as shown in Figure 2b. Therefore, an element size of 4 μm was used for the remainder of the study.

## 3. Results and Discussions

The three dimensional model described in the previous section was used to investigate the effect of laser system parameters, such as laser power, scanning speed, and beam focus on the depth, width, and shape of the channel. The effect of thermophysical properties, such as specific heat, thermal conductivity, and convection heat transfer coefficient on the channel geometry was also investigated. The system parameters and material properties investigated are provided in Table 1 and Table 2 respectively. All of the results presented in the report use the constant property values found in these tables unless stated otherwise. The material properties used to correspond to typical acrylic/PMMA properties from literature [32].

### 3.1. Effect of Laser Power and Unfocused Beam

The laser beam is said to be fully focused when the substrate is located at the focal point of the focusing lens. In focused beam laser ablation, the beam converges to its minimum radius value so that the spot size (i.e., the size of the area irradiated by the beam) is minimized. Thus the spot size can be controlled by adjusting the distance between the substrate and focal point using the following equation.

(9) $$d=\frac{\pi w_{0}^{2}}{\lambda }\sqrt{{\left(\frac{w}{w_{0}}\right)}^{2}-1}$$

**Figure 3:**
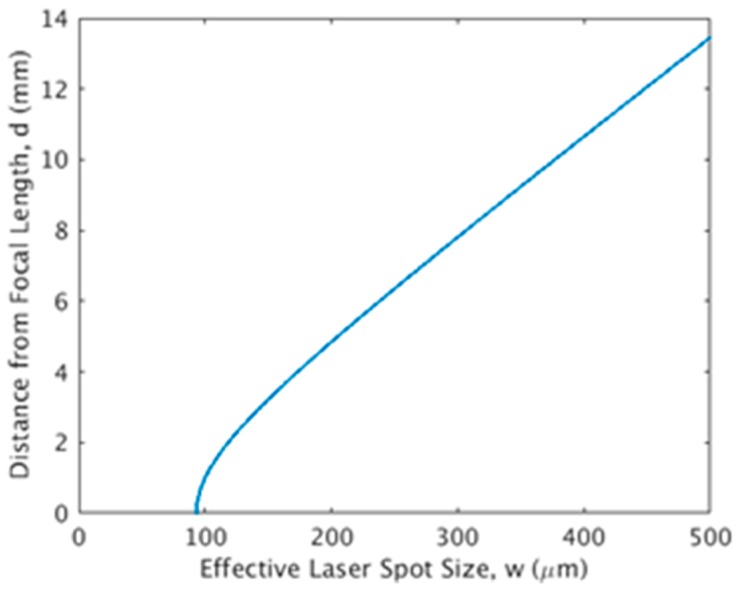
The laser spot size as a function of the distance between the focal point and the substrate.

Here, *w*_0_ is the spot size at the focal point. The value of *d* corresponds to a given spot size, *w* is shown in Figure 3. The spot size increases as the beam becomes less focused. This becomes less focused when the substrate is further from the focal point as seen in Figure 3. This means that the spot size of the laser beam on the material can effectively be set by adjusting the distance between the lens and the substrate, without needing to adjust the lens itself. 

**Figure 4:**
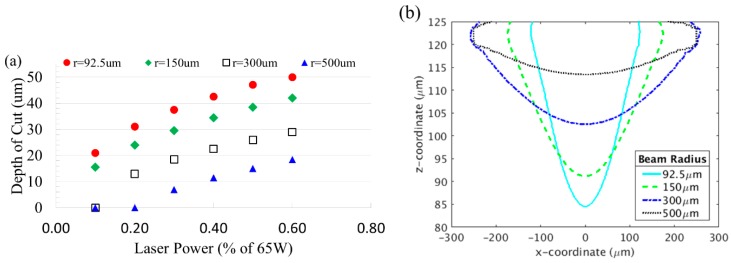
(**a**) Channel depth as a function of laser power for various spot sizes. (**b**) Shape of the channel cut out by 40% laser power with various laser spot sizes. Spot sizes are varied by considering focused and unfocused beam according to Equation (11).

The results of varying levels of focus, starting with a fully focused beam (spot size *w* = *w*_0_ = 92.5 µm), are shown in Figure 4 for various laser power settings. The results show that as the laser beam becomes less unfocused, channel width increased while depth decreased. This is due to the energy of the laser beam being distributed over a larger area, decreasing the intensity of the heat added at any given point. The decreased intensity prevents the heat from penetrating deeper into the material. For a given spot size, the depth of cut is not perfectly linear with the power. At lower power settings and larger spot sizes, the intensity of the heat was too low to remove material. Therefore there is a minimum laser power needed for creating a channel on the substrate.

Figure 4b shows cross-sectional profiles of the channels cut by laser at 40% laser power. The larger the spot size, the shallower the channel becomes. As the heat is spread out over a much larger area, increasingly shallow and wide channels are seen. By varying the spot size, either by changing the vertical distance between the laser and the material, or by varying the optical set up, the desired depth and width of channel can be achieved. It is also shown that the channel closely matches the shape of a Gaussian function at smaller spot sizes (92.5 and 150 µm). For smaller spot sizes, the laser beam is more focused with less loss of the impinged power at the surface. However, as the radius increases to 500 µm, the shape deviates from the Gaussian profile, especially at the edge of the top surface. 

### 3.2. Effect of Scanning Speed

**Figure 5:**
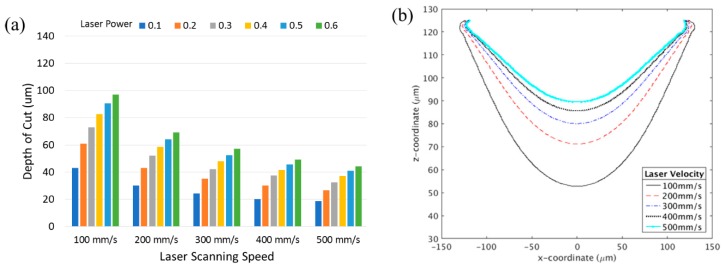
Effect of laser scanning speed on the laser ablation; (**a**) the depth of cuts for various laser scanning speed are presented with increasing laser power. (**b**) Channel depth as a function of laser power for various spot sizes. (**b**) channel shape for the different scanning speed with 40% of laser power.

During the laser machining, the speed of the laser is generally held fixed for consistency in results. However the scanning velocity of the laser beam during the ablation process directly determines the exposure time of the material. Thus, the laser speed has an impact on the total heat added, and by extension the depth of cut and other characteristics of the microchannel [27]. The results of varying the laser scan speed and fraction of laser power on the depth of cut are presented in Figure 5.

As the laser velocity was increased, the channel depth decreased. At higher laser scanning speeds, the total amount of energy imparted on the material is reduced, thus resulting in lower cut depths. At this point, it is important to note that the same depth can be found for various power and speed combinations. For example, a power setting of 10% and laser speed of 100 mm/s gives the same depth as setting the power to 20% with a laser speed of 200 mm/s. Laser scanning speed also contributes to the quality of the surface of the channel. For instance, the roughness of the cut has been reported to increase with decreasing laser scanning speeds [27]. The overall cross-sectional profile of the channel also varies with the speed as seen in Figure 5b. It is shown that the width of cut remains approximately the same, regardless of the scanning speed, while the depth varies. Thus, various aspect ratios of channel cross-section can be obtained simply by varying the laser speed.

### 3.3. Effect of Thermal Conductivity

**Figure 6:**
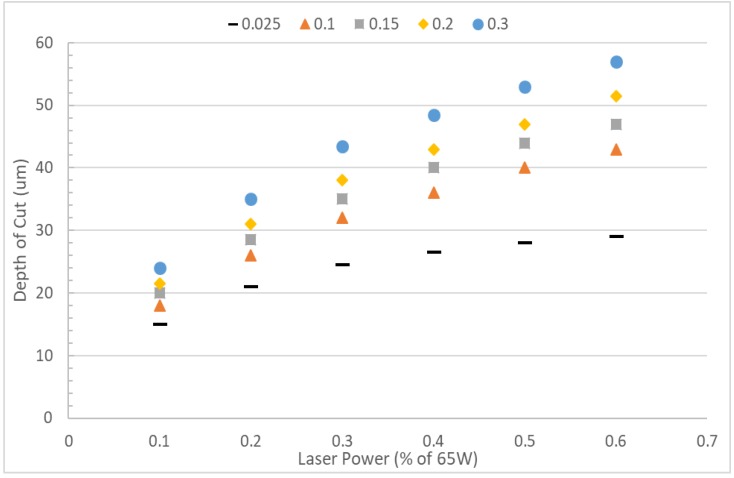
Depth vs. laser power for various thermal conductivity values. All material and system properties, other than thermal conductivity, are set to the values found in Table 1 and Table 2.

The effect of substrate thermal conductivity on the depth of cut is presented in Figure 6. Thermal conductivity values for polymers usually fall in the range of 0.1–0.3 W m^−1^ K^−1^ [37]. For instance, the thermal conductivity of PMMA is 0.19 W m^−1^ K^−1^. In laser ablation, heat is localized to a very small region of intense heat. In a material with poor thermal conductivity, heat is not able to spread far from the initially irradiated region. Figure 6 shows that higher thermal conductivity results in a deeper cut. Heat can easily spread further down into the material with higher thermal conductivity before it is ultimately removed by convection.

### 3.4. Effect of Specific Heat

The effect of the specific heat of substrates on the depth of cut was investigated for a range of constant specific heats (between 500–3000 J kg^−1^ K^−1^) as well as temperature dependent specific heat. Specific heat varies significantly for a material that undergoes a phase change. Temperature dependent specific heat was specified using the following equation adopted from [32].

(10) $$C_{p}=C_{p,solid}\left[1-H\left(T\right)\right]+\Delta h_{latent}D\left(T\right)+C_{p,vapor}H\left(T\right)$$

where *H(T)* is the smoothed Heaviside function centered at the vaporization temperature, and *D(T)* is a function used to distribute the latent heat of phase change across the phase change interval. Smoothed step functions were used when phase change occurs. 

**Figure 7:**
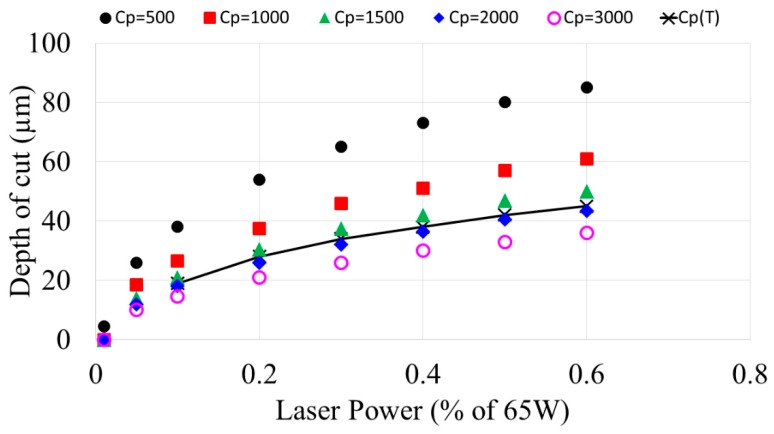
Channel depth for materials for a range of specific heats in laser ablation as a function of laser power.

Figure 7 shows that for constant lower specific heat values, the depth is much more sensitive to the effect of laser power. At higher laser powers, the effect is more pronounced. It can be noted that for a range of depths, multiple combinations of specific heat and laser powers are available. For instance, a material of specific heat 500 J/kg·K with a laser power of 6.5 W gives the same channel depth as a using 19.5 W laser power on a material with specific heat 1500 J/kg·K. This means that the same depth of cut is achievable across various types of substrate materials through adjusting the laser power. The depth of cut resulting from the phase dependent specific heat also shown in Figure 7. It shows that the constant specific heat assumption can overestimate the depth of cut by as much as 10%. This is significant for microfluidic applications, so it is imperative to consider temperature dependent specific heat in computer simulation of laser micromachining of microfluidic devices. 

### 3.5. Effect of Convective Heat Transfer Coefficients

**Figure 8:**
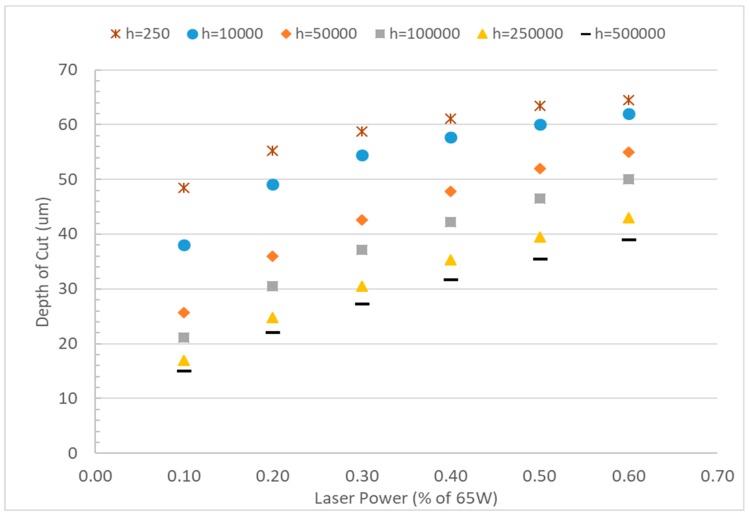
Depth vs laser power for various convection coefficients. Variation in the depth of cuts is observed for the higher convective coefficient.

The effect of convective heat transfer coefficients on the depth of cut was evaluated for a wider range of convection coefficients between 250 and 5 × 10^5^ W m^−2^ K^−1^. Typical forced convection heat transfer coefficient of 250 W m^−2^ K^−1^ was used to compare with the significantly higher values. Figure 8 shows that use of 250 W m^−2^ K^−1^ as heat convection coefficients cannot capture the variation of the channel for various laser power. The variation of depth due to increasing laser power becomes even more insignificant when lower convective coefficients were used (not shown). However, it has already been established previously that laser power has a significant effect on the depth of cut in laser ablation [21,25,29]. The depth of cut decreases with increased convection coefficient, regardless of the power settings. However, the depth of cut increases significantly with laser power at higher convection coefficients as well, which resembles the experimental observation of laser ablation. The larger heat convection coefficient accounts for not only the amount of heat removed by natural convection at the surface, but also the effect of additional heat escaping the bulk material. Heat escapes during melting and vaporization of the material due to laser treatment. 

## 4. Conclusions

This paper presents a mathematical model where high convective heat transfer coefficients were used to simplify the modeling of complex phase transitions and material removal processes found in laser microfabrication. The developed model was used to determine the effect of individual laser system parameters and material properties on the resulting fabricated channel. It was found that the laser system parameters such as power, beam focus, and scanning speed can be used to control and, eventually fine-tune the fabrication process for both channel width and height. The laser impingement spot size can be controlled by the level of focus of the beam. Similarly by selecting appropriate substrate materials, it would be possible to fabricate a channel with a desired width and depth using laser machining. This study provides the following conclusions:

1. The laser impingement spot size depends on the level of focus of the beam. Smallest spot sizes are achieved with a fully focused beam when material is located at its focal point, and spot size can be increased by increasing the distance between laser and material.

2. Larger laser spot size creates wider and shallower channels due to more scattered nature of the impinged energy.

3. The laser scanning speed mainly affects the depth with little to no impact on the width of the channel. If the laser scanning speed is increased, the depth of cut is decreased.

4. Substrates with higher thermal conductivity will have a deeper depth of cut for a given laser power and other system parameters.

5. The depth of cut increases as the laser power increases. Larger heat convection coefficient can be used to simplify the mathematical modeling of laser machining, to predict qualitatively the variation in depth as a function of laser power. 

6. The higher the specific heat of a substrate the lower the depth of cut for a given laser power. The use of temperature dependent specific heat is more appropriate for computer modeling compared to constant specific heat in laser micromachining.

## Figures and Tables

**Table 1:** Material Properties (for PMMA) used in the simulation [32].

Property	Units	Constant Value	Parametric Range
Thermal Conductivity, *k*	Wm^−1^K^−1^	0.19	0.019–3.8
Specific Heat, *C_P_*	Jkg^−1^K^−1^	1477	500–3000
Density, *ρ*	kg/m^3^	1180	-
Emissivity, *ε*	1	0.91	-
Absorption Coefficient (at 10.6 µm), *α*	m^−1^	1 × 10^6^	-

**Table 2:** System parameters used within the model.

Property	Units	Constant Value	Parametric Range
Laser Power Output, *I*_0_	W	65	-
Beam Radius, *w*_0_	um	92.5	92.5–500
Laser Velocity, *V_L_*	mm/s	381	50–500
Cut Length	mm	2	-
Convection Coefficient, *h*	Wm^−2^K^−1^	1 × 10^5^	2.5–5 × 10^5^
